# Copper/carbon nanotube composites: research trends and outlook

**DOI:** 10.1098/rsos.180814

**Published:** 2018-11-28

**Authors:** Rajyashree M. Sundaram, Atsuko Sekiguchi, Mizuki Sekiya, Takeo Yamada, Kenji Hata

**Affiliations:** CNT-Application Research Center, National Institute of Advanced Industrial Science and Technology (AIST), Central 5, 1-1-1 Higashi, Tsukuba 305-8565, Japan

**Keywords:** copper/carbon nanotube (Cu/CNT) composite, copper-substitute, composite homogeneity, Cu–CNT interfacial interaction, Cu and CNT structural control, Cu/CNT industrialization and application

## Abstract

We present research progress made in developing copper/carbon nanotube composites (Cu/CNT) to fulfil a growing demand for lighter copper substitutes with superior electrical, thermal and mechanical performances. Lighter alternatives to heavy copper electrical and data wiring are needed in automobiles and aircrafts to enhance fuel efficiencies. In electronics, better interconnects and thermal management components than copper with higher current- and heat-stabilities are required to enable device miniaturization with increased functionality. Our literature survey encouragingly indicates that Cu/CNT performances (electrical, thermal and mechanical) reported so far rival that of Cu, proving the material's viability as a Cu alternative. We identify two grand challenges to be solved for Cu/CNT to replace copper in real-life applications. The first grand challenge is to fabricate Cu/CNT with overall performances exceeding that of copper. To address this challenge, we propose research directions to fabricate Cu/CNT closer to ideal composites theoretically predicted to surpass Cu performances (i.e. *those containing uniformly distributed Cu and individually aligned CNTs with beneficial CNT–Cu interactions*). The second grand challenge is to industrialize and transfer Cu/CNT from lab bench to real-life use. Toward this, we identify and propose strategies to address market-dependent issues for niche/mainstream applications. The current best Cu/CNT performances already qualify for application in niche electronic device markets as high-end interconnects. However, mainstream Cu/CNT application as copper replacements in conventional electronics and in electrical/data wires are long-term goals, needing inexpensive mass-production by methods aligned with existing industrial practices. Mainstream electronics require cheap CNT template-making and electrodeposition procedures, while data/electrical cables require manufacture protocols based on co-electrodeposition or melt-processing. We note (with examples) that initiatives devoted to Cu/CNT manufacturing for both types of mainstream applications are underway. With sustained research on Cu/CNT and accelerating its real-life application, we expect the successful evolution of highly functional, efficient, and sustainable next-generation electrical and electronics systems.

## 1. Introduction

Copper is an indispensable material in our modern electricity- and electronics-driven society. It is the most electrically conducting non-precious metal (5.8 × 10^5^ S cm^−1^ at 27°C) [1] and the best thermal conductor among metals (401 W m^−1^ K^−1^ at 27°C) [2]. Further, copper has a high current-carrying capacity [3], is strong, ductile, workable, and resistant to corrosion and creeping [4]. This combination of stellar attributes makes copper the material of choice for a range of applications. Due to its exceptional electrical properties, copper is used in electrical power cables and wiring, generators, motors, transformers, data and phone wires, and as connectors in electronics. As a superior heat transporter, copper is used as heat-sinks in electronics and heat exchange equipment, such as vehicle radiators. According to the Copper Alliance [5], ‘one tonne of copper brings functionality in 40 cars, powers 60 000 mobile phones, enables operations in 400 computers, and distributes electricity to 30 homes’. As a robust durable metal, copper dominates industrial components, such as bearings, gears and turbine blades. However, copper is heavy (density 8.9 g cm^−3^ [2]) and soft. In electronics, copper interconnects disintegrate at high currents and delaminate from Si chips due to thermal expansion arising from heat build-up [6]. This leads to device failure, which is exacerbated in downsized interconnects and high-power devices.

Next-generation macro and microscale applications aiming for higher functionality, efficiency and sustainability demand materials outperforming copper. There is a growing need to replace heavy copper electrical and data wiring in vehicles with lighter alternatives for improved fuel efficiency. An average mid-size automobile contains about 22.5 kg of copper, while electric and hybrid vehicles could contain higher levels of copper [4]. Trains and commercial aircraft contain a few tons of copper [4]. Replacing approximately 2 tons of Cu wiring in a commercial aircraft with a material two-thirds of the weight translates to 25 000 tons of fuel savings and 78 000 tons of CO_2_ emission cuts per year [7] (https://www.lufthansagroup.com/fileadmin/downloads/en/responsibility/balance-2017-epaper/#0 (accessed 26 April 2018)). The electronics industry on the other hand requires interconnects with higher current and heat stabilities and better heat-sinks than copper to keep up with rapidly miniaturizing devices of growing complexity and power consumption [8].

Cu/carbon nanotube (CNT) composites that merge copper with CNTs are touted to fulfil the growing need for Cu substitutes [8–17]. CNTs are expected to play two roles in Cu/CNT. (i) Made of carbon, CNTs act as weight reducers, rendering the composites lighter. (ii) CNTs could transmit their own exceptional nanoscale multifunctional properties to Cu to yield composites with superior performances. It is well known that individual CNTs are strong, ballistically transport electrons and are excellent thermal conductors [18,19]. Indeed, CNT addition to Cu is reported to lead to improved mechanical, electrical and thermal properties [8–17], demonstrating the promise of Cu/CNT.

CNT macromaterials and other metal-matrix/CNT composites (e.g. Al/CNT) are additionally explored prospective Cu alternatives besides Cu/CNT. However, these materials are unlikely to match the promise of Cu/CNT, specifically in applications requiring high electrical conductivities. Individual CNTs are nanoscopic and too small for practical application, although they surpass copper performances. More practically useful macromaterials composed of CNT assemblies have been fabricated but their performances are inadequate. For instance, CNT macromaterial electrical conductivities are at least an order of magnitude lower than that of copper, limiting their practical application [18,19]. In addition, CNT macromaterials also present issues with regard to integration into existing electrical and electronics systems. Standard joining methods (soldering, crimping, etc.) are inapplicable to the purely carbon-based nanotube assemblies. Among metal-matrix/CNT composites prevalently researched, Al/CNT composites compete closely with Cu/CNT as Al is lighter and cheaper than copper. However, by itself, Aluminium has a lower melting point and higher propensity for oxidation than copper, rendering Cu-based composites more promising, especially for applications requiring thermal stability [2,20].

**Figure 1.**
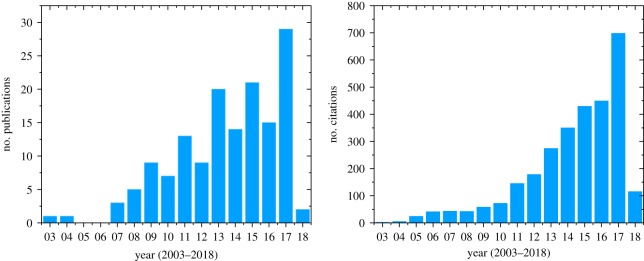
Publication trends of peer-reviewed articles on CNT–Cu composites (source: Web of Science, search terms: CNT-copper composite and Carbon-nanotube copper composite).

Due to Cu/CNT's unmatched promise and potential impact as a Cu alternative, research interest on these materials from both academic and industrial communities has been increasing. Over the years, the number of publications and their citations on Cu/CNT development has steadily increased (figure 1). On account of the research community's sustained interest in Cu/CNT, extensive review articles have been published at regular intervals [8–17]. However, a unified view on the field's development so far and its likely future course are missing. This mini-review aims to present a cohesive picture of Cu/CNT research progress to date to determine the material's real ability to substitute copper (§2). Further, we will pinpoint challenges and solutions to enable Cu/CNT's real-world application (§3).

## 2. Research trends and milestones

**Figure 2.**
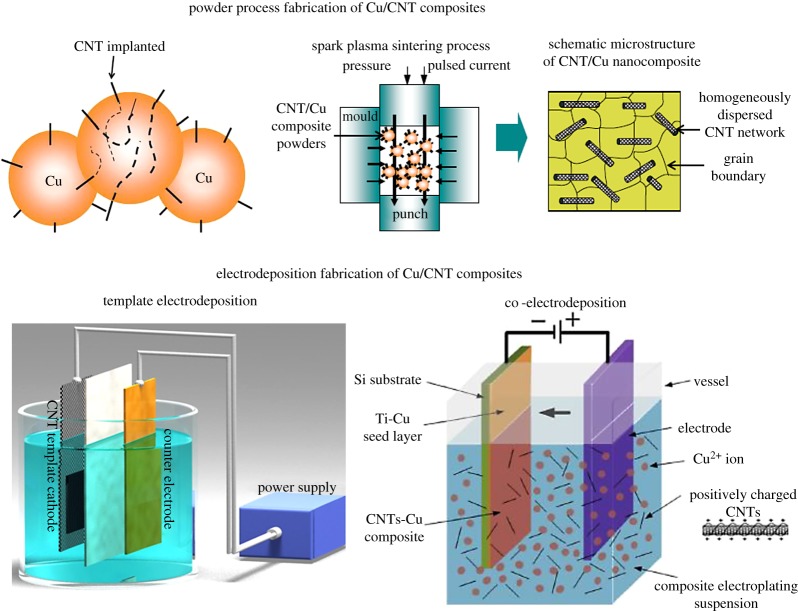
The two major CNT/Cu fabrication routes: powder-processing [21] and Cu electrodeposition with figures on left and right representing template electrodeposition (altered from reference [6]) and co-electrodeposition [22], respectively. Images from references 21 and 22 reproduced with permission.

Two major approaches have been developed for Cu/CNT fabrication—(1) powder-processing and (2) electrochemical deposition (figure 2). Powder-processing (approach 1) usually involves making Cu/CNT nanocomposite powders by mixing CNT and Cu powders, followed by compaction. Common mixing methods are ball/attrition milling [23–29] and ultrasonication [30–32]. Alternative methods (also used in combination with milling/ultrasonication) to make nanocomposite powders include using CNT powders for electroless Cu deposition [33–39] and molecular-level mixing with copper salts [21,31,40–47]. Usual compaction methods are spark plasma sintering (SPS [28–32]), isostatic pressing [25,43,47], high-pressure torsion (HPT) [45,48–50], forging [36,37], etc. In approach 2, Cu is electrochemically deposited at CNT template cathodes by reducing Cu ions in solutions [3,6,51–67]. Alternatively, CNTs and Cu are co-deposited from dispersions of charged nanotubes in Cu-ion solutions [22,68–70]. In addition to these two major approaches, other methods, such as physical vapour deposition [71], magnetron sputtering [72], etc. have also been recently used for composite fabrication. In the following sub-sections, we examine the development and achievements of the two major fabrication approaches.

### 2.1. Powder-processing Cu/CNT composites

Powder-processing has been used to make the earliest composites (in 2001 [23]) as research on metal-matrix/CNT composites evolved from CNT-ceramics composites studies [9], borrowing processing techniques, such as milling, sintering, etc. In general, powder-processing has mainly focused on improving mechanical properties of Cu by adding CNTs as reinforcing agents, although other properties (electrical and thermal) have been explored. Over nearly two decades of Cu/CNT research, powder-processed composites with higher strength, modulus, hardness and wear properties than Cu have been demonstrated. Progress has been made in inventing mixing methods to achieve homogeneous CNT/Cu distribution and in scaled-up fabrication of application-ready structures, such as macroscopic wires.

The first powder-processed composites were fabricated by ball milling multi-walled carbon nanotubes (MWCNTs) and Cu powders followed by isostatic pressing and isothermal sintering [23]. The composites with up to 12 vol% CNTs demonstrated better wear properties and hardness than copper. However, physically mixing CNTs and Cu powders results in poor adhesion between CNTs and Cu due to density differences of the two materials (less than 2.0 g cm^−3^ for CNTs [18,19] versus 8.9 g cm^−3^ for Cu [2]). The lighter CNTs remain on the Cu particle surface instead of embedding within. This leads to non-uniform composites after compaction, with agglomerated CNTs poorly dispersed in the matrix. To address this issue, CNTs made heavier by coating with other metals (such as Ni [23]) are used for the mixing stage. However, other mixing methods were required to improve CNT–Cu interaction to ensure uniform CNT–Cu distribution without a third element.

**Figure 3.**
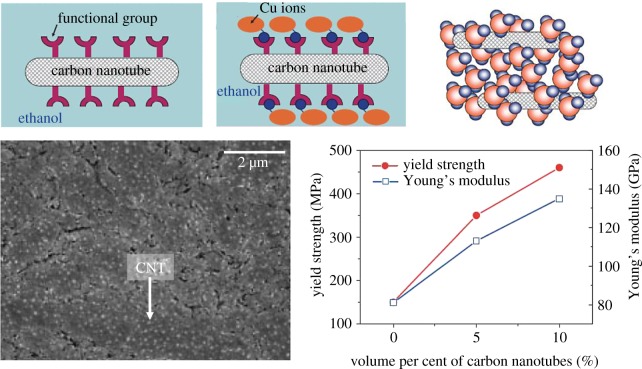
Homogeneous Cu/CNT with superior mechanical properties than Cu fabricated by molecular mixing [21]. Images reproduced with permission.

A novel molecular-level mixing method was developed to enhance CNT–Cu interaction (figure 3) [21,31,40–47]. In this method, suspensions of CNTs with anionic functional groups are mixed with Cu^2+^ salt solutions, which encourages electrostatic interaction between the two entities (rather than merely physical adhesion). The CNT/copper salt solutions are dried and calcined to make copper-oxide/CNT composite particles, which are then reduced, usually by H_2_-annealing to CNT-embedded Cu nanocomposite powders. These nanocomposite powders are then compacted by SPS [21,42,44] or isostatic pressing and thermal sintering [43]. The first molecular-level mixing composites with 5 and 10 vol% MWCNTs showed yield strengths 2–3 times that of Cu along with a higher Young's modulus than Cu (figure 3) [21]. This remarkable reinforcement was attributed to high load-transfer efficiency due to improved CNT–Cu bonding. Xue *et al*. showed that the yield and tensile strengths of Cu/MWCNT_(5 vol%)_ composites prepared by molecular-level mixing are higher than their ball-milled counterparts [44]. Molecular-level mixed composites also show a lower coefficient of thermal expansion (CTE) (of 12.1 ppm) than Cu (17 ppm) [42] and a lower mismatch with Si CTE (of 3–5 ppm). This indicates the Cu/CNT's potential as a better interconnect material than copper for electronics with higher thermal stabilities. The lower CTE mismatch with Si (the usual substrate in electronics) is critical for interconnect materials, to avoid their thermal expansion-driven delamination from the chip. Mendoza *et al*. demonstrated the applicability of molecular-level mixing to single-walled (SW) CNTs [43]. Their Cu/SWCNT_(10 vol%)_ composites showed an increase in both hardness (42% compared to Cu) and electrical conductivity (three times higher at 80 K).

Besides achieving homogeneous CNT/Cu distribution, powder-processing has been successful in fabricating composites in application-ready configurations, such as wires. Arnaud *et al*. [30] report 1.5 m long Cu/CNT wires made by powder-processing with diameters similar to industrial copper wires (0.2–1 mm). The Cu/DWCNT_(0.5 vol%)_ wires were produced by ultrasonication mixing, followed by SPS and wire drawing and showed tensile strengths greater than Cu and room temperature electrical conductivities similar to Cu.

Despite achievements in making possible the fabrication of homogeneous composites with high performances, powder-processing poses several limitations.

**Figure 4.**
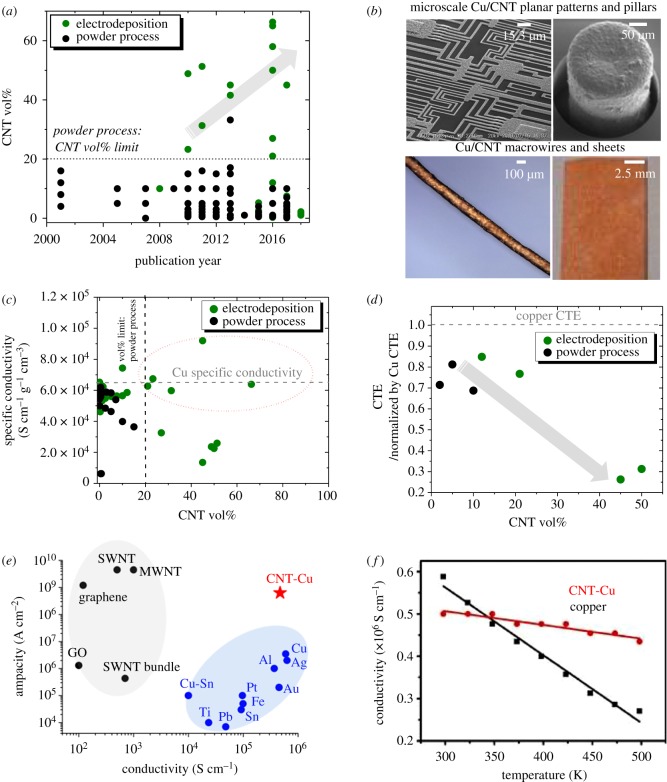
Advantages of electrodeposition versus powder-process Cu/CNT fabrication: (*a*) reported composite CNT vol% trend (over years), (*b*) various configurations of electrodeposited Cu/CNT ([3,59,60,63]: clockwise from top-left), (*c*) specific conductivity and (*d*) CTE versus CNT vol%. Plots depicting the (*e*) current stability [3] and (*f*) conductivity stability with temperature of electrodeposited composites [3]. Images in (*b*), (*e*), and (*f*) are reproduced with permission from references [3,59,60,63].

(1) The CNT vol% routinely achieved in powder-processed composites is less than 20 vol% (figure 4*a*). Therefore, most composites show densities nearly equivalent to Cu with no weight reduction advantages. Guirderdoni *et al*. attempted to include approximately 33 vol% CNTs in their powder-processed composites [32]. However, the samples were found to lack CNT distribution homogeneity and comprised areas with and without nanotubes.
(2) Powder process precludes CNT alignment in composites as the methodology unavoidably involves a mixing step. Additional post-production steps have been applied to align CNTs, such as high-ratio differential speed rolling [24], die stretching [36,37] and magnetic field application [35]. Although these studies demonstrate that CNT alignment benefits Cu/CNT mechanical, thermal and electrical properties, most samples contain less than 10 vol% nanotubes. Increasing CNT vol% with alignment and uniform dispersion in Cu matrix is necessary to achieve lightweight composites with high performances, specifically electrical and thermal conductivities.
(3) Powder-processing is incompatible to make microscale composite structures required for electronic devices, such as vias or horizontal interconnects on Si substrates. Most powder-processed samples are macroscopic pellets [43], or cylinders and wires [30].

### 2.2. Electrodepositing Cu/CNT composites

Copper electrodeposition of CNT templates from Cu^2+^ salt solutions emerged as an alternative Cu/CNT fabrication method, overcoming the limits of powder-processing. Electrodeposition has made lightweight composite fabrication possible by allowing inclusion of higher CNT vol% than powder-processing (figure 4*a*). Further, electrodeposition allows for easier CNT alignment control than powder process, and composites with CNTs in networks (no alignment) [51], cross-ply [62] and unidirectional alignment [55,57,59,60] have been fabricated. In addition, electrodeposition affords Cu/CNT fabrication at various scales and in different configurations (figure 4*b*). Both microscale Cu/CNT, such as pillars [52,59] and patterns [3,6,60] on Si substrates (for device application) and macroscale samples, such as sheets/films [55,57,62,65] and wires [53,54,56,63,64,66,67] (for power lines, motor windings, etc.) have been reported.

Developing structurally well-regulated CNT templates and establishing processing/deposition protocols suitable for these templates have been critical for electrodeposition to emerge as a viable composite fabrication approach. Synthesizing CNT templates is now fairly well-established. CNT materials of various configurations, such as macro wires and sheets or micropillars with controlled CNT orientation and structures are now readily available [73–75] and even produced commercially (https://www.veelotech.com/products-1/ (accessed 16 May 2018); http://www.nanocomptech.com/ (accessed 16 May 2018)). In terms of processing, strategies to improve CNT template wetting by the electrolyte have advanced as typical aqueous copper salt electrodeposition solutions (like CuSO_4_/H_2_SO_4_) are unsuited for hydrophobic CNT templates. These strategies involve either (i) increasing CNT template hydrophilicity or (ii) modifying Cu electrolyte to suit hydrophobic CNTs.

(i) *Increasing CNT template hydrophilicity:* CNTs have been functionalized with oxygen-containing groups to improve hydrophilicity and, thereby, wetting by aqueous Cu electrolytes. Usual functionalization methods are anodization [54,66], heat-treatment in oxygen atmosphere [66], etc. Besides facilitating Cu deposition, using oxygen-functionalized templates is also reported to improve CNT–Cu interaction in the composites, leading to better properties than composites obtained from non-functionalized templates. For example, Cu/CNT wires obtained by Cu electrodeposition after template anodization are seen to exhibit higher tensile strengths and electrical conductivities than Cu/CNT wires made without the anodization pre-treatment [54].
(ii) *Modifying Cu electrolyte to wet hydrophobic CNTs:* Alternative electrolytes, such as organic solutions of Cu salts capable of wetting hydrophobic CNTs and infiltrating templates have been developed to facilitate Cu deposition [3,6,59,60,63,64,67]. Mainly, copper acetate in acetonitrile is used for copper deposition. However, since organic solutions result in slow and insufficient deposition [64], the method has been used as a seeding step and supplemented by a second seed-growth step using CuSO_4_/H_2_SO_4_ electrodeposition. This two-stage process has shown success in fabricating Cu-matrix composites embedding a high vol% of CNTs (45–50 vol%) at both microscale (planar and vertical interconnects) [3,6,59,60] and macroscale (sheets and wires) [3,63,64,67]. The two-stage process overcomes the limitation of CNT template direct aqueous electrodeposition that invariably leads to Cu-coated composites (laminates or core–shell structures) [53–57,61,62,65,66] regardless of template functionalization.

The major achievement of electrodeposition has been in revealing the potential of Cu/CNT as a Cu alternative by producing lightweight composites with electrical, thermal and mechanical performances rivalling that of Cu. As mentioned before, electrodeposition succeeded in breaking the CNT vol% limit posed by powder-processing. High CNT vol% means composites markedly lighter than copper, which despite the high carbon content, are seen to perform at par with copper. Randeniya *et al*. [53] and Xu *et al*. [54] achieved Cu/CNT wires one fifth to half as light as copper by electrodepositing CNT wires. The wires showed electrical conductivities approximately 1.8–3.0 × 10^5^ S cm^−1^ (versus 5.9 × 10^5^ S cm^−1^ for Cu) and high tensile strengths of approximately 500–800 MPa (versus approx. 220 MPa for copper). Low densities and high performances competitive to Cu translate to specific properties (density-normalized properties) exceeding that of Cu. As seen in figure 4*c*, electrodeposited composites with high CNT vol% show specific electrical conductivities surpassing Cu. On the other hand, the specific values of powder-processed composites remain below copper with the low CNT vol%. Besides weight reduction, high CNT vol% composites are necessary for other properties, such as heat stability (especially, low coefficients of thermal expansion, CTE). Low CTE values closer to Si (approx. 2.6 ppm [76] versus approx. 17 ppm for Cu [2]) are sought after for device interconnects to avoid expansion-induced delamination from substrates. Both electrodeposited [6,22,59] and powder-processed [42,77] composites show reduction in CTE compared to Cu (figure 4*d*). The CTE is lowered in Cu/CNT because Cu expansion is offset by CNTs, which by themselves show low/negative thermal expansions (due to shrinking in-plane sp^2^C–sp^2^C bonds) [6]. However, electrodeposited composites with a higher CNT vol% show much lower CTE values than the powder-processed samples (figure 4*d*). For example, electrodeposited composites with 45–50 vol% CNTs [6,59] show CTE values of approximately 5 ppm K^−1^ that match with Si. These low CTE values were achieved in combination with thermal conductivities similar to Cu (approx. 395 W m^−1^ K^−1^) [6,59], which is beneficial to minimize heat build-up.

Electrodeposited composites also exhibit higher current stability and temperature-stable electrical conductivities compared to copper, which further corroborate Cu/CNT's potential as Cu substitutes, especially for electrical applications. Current stability is the ability of a conducting material to carry high currents without damage and is quantified in terms of current-carrying capacity (CCC) and lifetime. High CCC and lifetimes are highly desirable attributes for electrical conductors, specifically interconnects in downsized and high-power electronics. Electrodeposited Cu/CNTs have demonstrated current stabilities greater than Cu in both micro [3] and macroscopic structures [57,63]. Cu/CNT microlines with CCCs 100 times that of Cu [3] have been reported (figure 4*e*), while macrosheets [57] and wires [63] with 36% and 28% increase in CCC versus Cu, respectively, have been demonstrated. In terms of lifetime, Chai & Chan [52] show a fivefold increase in lifetime for vertical Cu/CNT via-interconnects versus Cu. Recently, Subramaniam *et al*. [60] confirmed lifetime increase in planar multitier Cu/CNT interconnects compared to Cu. The improved Cu/CNT CCC and lifetime (versus Cu) are attributed to nanotubes suppressing Cu electromigration (movement of Cu along electric current) by increasing Cu diffusion activation energy [3].

Besides current stability, electrical conductors used for applications involving high operating temperatures (motor windings, high-power device interconnects, etc.) require stable electrical conductivity versus temperature. Normally, electrical conductivity of Cu decreases with temperature due to increased electron scattering, resulting in a large temperature coefficient of resistance (TCR, approx. 3.9 × 10^−3^ K^−1^) [78]. Adding CNTs (which by themselves show small TCR values) to Cu results in TCR reduction and both macro and microscopic Cu/CNT show TCR less than Cu [3,63]. For example, Cu/CNT macrosheets and wires with TCR values 50–80% that of Cu [57,63] have been fabricated. At the microscale, planar and vertical interconnects with TCR one tenth [3] and half [59] that of Cu, respectively, have been demonstrated. As an added benefit, TCR suppression can lead to composite electrical conductivities greater than Cu at high temperatures (figure 4*f*). Subramaniam *et al*.'s planar Cu/CNT interconnects show electrical conductivities higher than Cu above 80°C [3], which are typical operating temperatures in high-power electronics (figure 4*f*).

In addition to CNT template–Cu electrodeposition, co-electrodeposition of CNTs and Cu has also been used to fabricate Cu/CNT [22,68–70]. The main advantage of co-electrodeposition over template electrodeposition is its compatibility with device fabrication. Unlike template electrodeposition, co-electrodeposition does not require CNT synthesis/transfer onto Si chips involving high-temperature methods (like chemical vapour deposition). To co-deposit Cu and CNTs, a Cu^2+^ electrolyte solution with dispersed CNTs is used. Dispersing sufficiently deaggregated CNTs in the electrolyte to achieve uniform CNT/Cu mixing in the composite is the key issue in co-electrodeposition. To enhance CNT deaggregation, additives or CNT treatments have been used. For example, An *et al*. [22] used electrostatic repulsion between positively charged CNTs obtained by polyelectrolyte treatment, while Feng *et al*. [69] used nanodiamond particles as additives in the electrolyte. Co-electrodeposited composites show electrical conductivities comparable to that of copper and improvements in mechanical properties, such as strength, Young's modulus and hardness [22,68–70]. However, the maximum CNT vol% obtained in co-electrodeposited composites is approximately 21 vol% [22], which is less than that achieved by template deposition.

**Figure 5.**
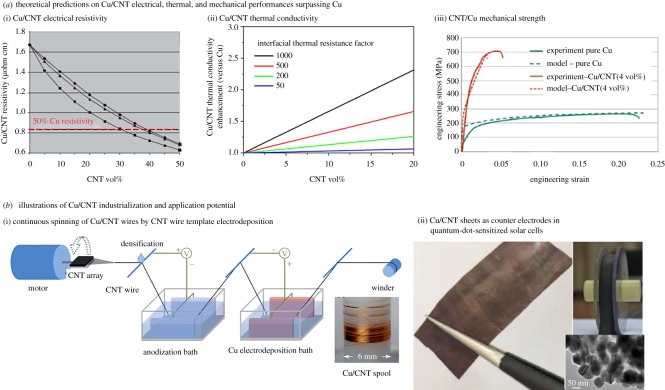
(*a*) Potential of Cu/CNT to surpass Cu performances: theoretical studies predicting Cu/CNT electrical resistivity (i) [79], thermal conductivity (ii) [80] and mechanical strength (iii) [81] greater than Cu. (*b*) Examples of Cu/CNT industrialization and application: continuous production by electrodeposition of strong lightweight high electrical conductivity Cu/CNT wires (i) [54] and application of continuously fabricated Cu/CNT sheets as high-efficiency counter electrodes in quantum-dot solar cells (ii) [82]. Images reproduced with permission.

Although high-performance composites with high CNT vol% that show promise as Cu alternatives have been fabricated (by template electrodeposition), Cu/CNT performances fall well below expectations. According to effective-medium model calculations, electrical conductivities 2 × Cu can be achieved in composites with greater than 30–40 vol% defect-free individual CNTs aligned along bias direction embedded in a Cu-matrix (figure 5*a*(i)) [79]. Similarly, atomistic simulations predict high thermal conductivities (>Cu) for composites that embed aligned minimally aggregated CNTs in Cu with a large CNT–Cu interface (figure 5*a*(ii)) [80,83]. Experimentally obtained values contradict these theoretical predictions and the highest observed composite electrical and thermal conductivities with 45 vol% aligned CNTs well-dispersed in Cu-matrix are less than or only comparable to Cu [3,6,59,63]. Encouragingly, the mechanical strengths of the composites exceed that of copper in agreement with modelling data (figure 5*a*(iii)) [81]. Therefore, the full potential of Cu/CNT in terms of overall electrical, thermal and mechanical properties is yet to be realized. In the next section, we will identify challenges that need addressing and propose directions for realizing and harnessing the true potential of Cu/CNT.

## 3. Cu/CNT research: goals, challenges and future

The research community's long-term goal is to replace copper with Cu/CNT that is lighter and with superior electrical, mechanical and thermal properties in applications ranging from data and electricity cables to interconnects. There are two grand challenges in achieving this goal—(1) fabricating Cu/CNT outperforming Cu as per theoretical predictions (figure 5*a*), and (2) enabling lab-to-market transition of Cu/CNT by industrialization and facilitating real-world utilization (figure 5*b*). Research directions to solve specific issues in each of the two grand-challenges are discussed in §§3.1 and 3.2.

### 3.1. Fabricating Cu/CNT outperforming Cu

**Figure 6.**
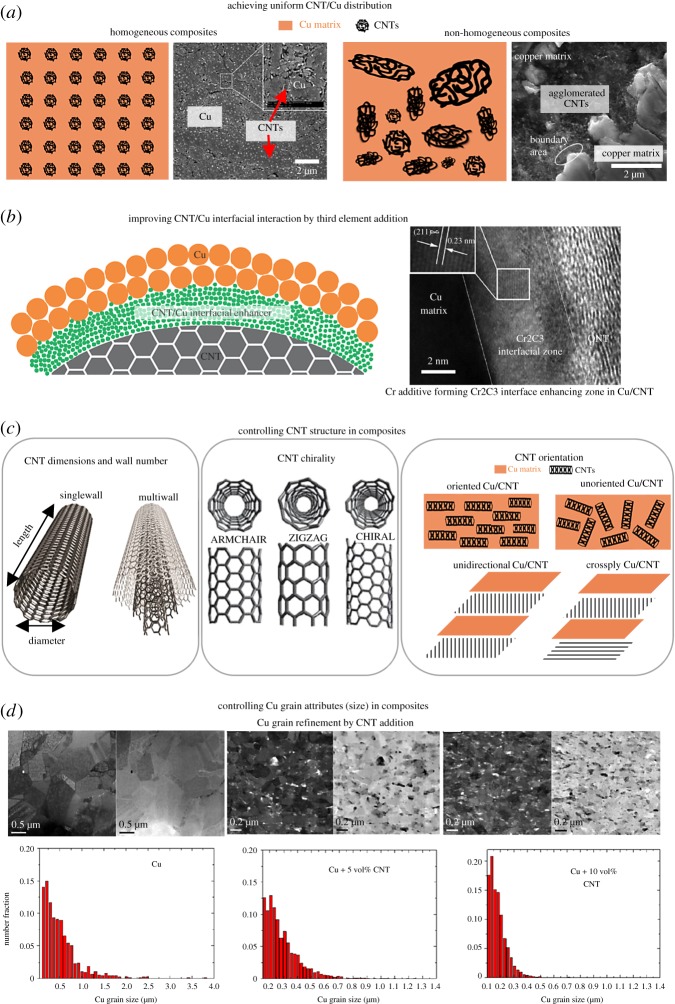
Summary of challenges in fabricating Cu/CNT outperforming copper. (*a*) Challenge 1: Achieving uniform CNT–Cu distribution. The figure depicts homogeneous and non-homogeneous composites with schematics and example SEM images from literature (left: [42], right: [35]). (*b*) Challenge 2: Improving CNT–Cu interfacial interaction with additives. A schematic of an interfacial enhancer included at the CNT–Cu interface is provided. An example research work from literature using an interfacial enhancer is included (TEM image from [84]). Challenge 3: Controlling CNT and Cu-matrix attributes. To summarize this challenge, (*c*) the various CNT attributes, to be controlled are illustrated (reproduced/altered from [62,85,86]), and an example of Cu-grain size control in Cu/CNT from literature is provided [87]. Images reproduced with permission.

The first grand challenge in Cu/CNT research is to fabricate composites with a high nanotube vol% as well as a combination of mechanical, thermal and electrical performances surpassing Cu. Theoretical studies that predict Cu/CNT to surpass performances (figure 5*a*) assume an ideal composite [79–81,83]. *The ideal composite consists of a continuous Cu matrix embedding a high vol% of individual unidirectionally aligned CNTs spatially distributed uniformly throughout. The CNT–Cu interactions in the ideal composite are assumed to allow for superior electron/phonon transport (*figure 5*a*(i)(ii)) *and for CNTs to mechanically reinforce the metal matrix* (figure 5*a*(iii)) *improving electrical, thermal and mechanical properties*. However, there is a mismatch between the ideal composite and experimental samples, and the observed Cu/CNT performances have not met expectations. We identify three issues to be addressed to reduce the mismatch between ideal and real-world composites to achieve Cu/CNT that outperforms Cu:

(1) achieve uniform CNT and Cu distribution at high CNT vol% (figure 6*a*),
(2) ensure effective CNT–Cu interaction (figure 6*b*), and
(3) control CNT and Cu-matrix attributes (figure 6*c*,*d*).

Below, we propose research directions to address each issue in the context of efforts already reported in literature.

#### 3.1.1. Achieve uniform CNT and Cu distribution at high CNT vol%

The main difficulty with achieving uniform CNT and Cu spatial distribution is phase separation due to lack of interaction between individual nanotubes and copper. Phase separation arises from fabrication issues and manifests differently in powder-processed and electrodeposited composites. The issues and solutions for minimizing phase separation and improving CNT and Cu distribution for both fabrication methods are discussed below.

**(*a*) *Achieving uniform CNT and Cu distribution by powder-processing:*** Phase separation manifests as localized CNT agglomerations (figure 6*a*), aggravated with increasing CNT vol% in powder-processed composites [32,35]. The CNT agglomerations increase sample porosity and become the ‘weakest link in the chain’, accumulating local stress and increasing electron and phonon scattering. Consequently, powder-processed composites with localized CNT aggregations show deteriorated properties. Cho *et al*. [88] note CNT aggregation as the key cause for lowered thermal conductivity (versus Cu) in composites with at least 5 vol% CNTs. In contrast, their composites with uniformly distributed 3 vol% or less CNTs show slightly higher thermal conductivities than Cu. Similar reductions in powder-processed composite hardness, strength and wear properties versus CNT vol% ascribed to nanotube agglomeration have been observed widely. Phase separation in powder-processed composites is mainly seen in samples obtained by physical mixing, which ignores Cu versus CNT density differences and relies on weak van der Waals forces to induce CNT–Cu interaction.

Instead of physical mixing, molecular mixing (involving blending CNTs and copper salts) [21,31,40–47] that seeds Cu on nanotubes through stronger electrostatic interactions enhances homogeneity and properties of powder-processed composites (as seen in §2.1). The nanotubes are usually functionalized covalently or non-covalently to acquire a negative charge to interact with positively charged copper in salts. Non-covalent functionalization shows more promise for attaining property improvement with composite homogeneity by molecular mixing than covalent functionalization. Covalent CNT functionalization degrades the nanocarbon lattice (especially at the Cu-matrix/nanotube interface), which is disadvantageous for composite electron/phonon transport properties. Kim *et al*. [42] observed a 40–50% drop in thermal conductivity (versus Cu) in molecular mixed Cu/MWCNT, ascribed to phonon dissipation at functionalized nanotube/Cu interface. The nanocarbon lattice degradation also renders covalent functionalization incompatible for processing Cu/CNT with few-walled/single-walled nanotubes. On the other hand, by using non-covalently functionalized CNTs for molecular mixing, Mendoza *et al*. [43] fabricated SWCNT composites with electrical conductivity (at 80 K) and hardness better than copper. However, the effect of non-covalent nanotube functionalization on other composite properties (mechanical strength, thermal conductivity, CTE, CCC, etc.) are unknown. Besides molecular mixing, electroless Cu coating of functionalized CNTs [34,38,39] in solution have been used as a Cu seeding step (to supplement or replace physical mixing). However, these alternative seeding approaches have used only covalently functionalized CNTs so far. The use of non-covalently functionalized CNTs for these alternative Cu-seeding methods needs to be explored toward making homogeneous Cu/CNT with high comprehensive performances.

**(*b*) *Achieving uniform CNT and Cu distribution by template electrodeposition:*** CNT–Cu phase separation manifests as [CNT-core/Cu-sheath] structures in composites fabricated by aqueous-medium Cu electrodeposition of CNT templates [53–57,61,62,65,66]. Alternative protocols using organic solutions of Cu^2+^ as electrolytes (two-stage electrodeposition [3,6,59,60,63,64,67]) have been successful in obtaining Cu-matrix composites instead of core-sheath structures (as seen in §2.2). These composites have so far shown the best combination of properties reported, i.e. highest CNT vol% and maximum observed electrical and thermal conductivities as well as current and heat stabilities (in terms of CCC, TCR and CTE). However, in the two-stage electrodeposited samples, CNT bundles rather than individual CNTs are embedded in the Cu matrix, leading to the less-than-predicted [79,80,83] electrical and thermal conductivities. CNT bundling reduces CNT–Cu interfacial area and, hence, suppresses nanotube contribution to composite performance. Currently, there are no reports for minimizing CNT bundling in composites obtained by two-stage electrodeposition. To minimize CNT bundling for composite performance enhancement, we propose using functionalized (preferably non-covalently) low-density CNT templates for two-stage electrodeposition.

#### 3.1.2. Ensure effective CNT–Cu interfacial interaction

The second issue to be addressed to achieve composite performances better than Cu is to ensure effective CNT–Cu interfacial interaction. By default, interfacial interaction is poor because CNTs and copper show poor affinity to each other. Cu, with its fully filled d orbitals, does not chemically interact with carbon. Further, Cu does not wet CNTs because of the high mobility of Cu atoms on CNTs [89], large surface energy difference between the two materials (Cu: approx. 1800 mJ m^−2^ [90] versus CNTs: approx. 30–45 mJ cm^−2^ [91]), and the high propensity of the materials to self-aggregate [92]. However, enhancing CNT–Cu interaction is critical for improving stress transfer and electron/phonon transport through composites. Improving CNT–Cu interaction can also be expected to aid CNT and Cu distribution uniformity (in addition to discussion in §3.1.1). To improve CNT–Cu interfacial interaction, several reports have attempted including an additive to the interface that strongly associates with both Cu and CNTs (figure 6*b*). Two classes of CNT–Cu interfacial interaction-enhancing additives have been identified—(a) oxygen and (b) carbide-forming metals.

**(*a*) *Oxygen as CNT–Cu interfacial interaction enhancer:*** Oxygen inclusion is usually accomplished by using nanotubes with oxygenated surface functional groups for composite fabrication. Both theoretical and experimental studies reveal the advantages of including oxygen to the CNT–Cu interface. Density functional theory calculations [93] show that Cu adsorbs more strongly to CNTs with oxygenated functional groups than to CNTs without these functional groups. For example, Cu adsorption binding energy is 1.37 eV for CNTs with –COOH groups versus 0.53 eV for neat CNTs. In experimental studies, Kim *et al*. [94] note that interfacial oxygen (on functionalized nanotube surfaces) improves composite strengths in molecularly mixed composites. The strength increase is attributed to stronger CNT–Cu interfacial bonding through oxygen, which leads to better load-sharing, allowing nanotubes to function more effectively as reinforcing agents. Further, composite homogeneity was also observed to increase as surface functional groups reduced nanotube agglomeration during fabrication.

**(*b*) *Carbide-forming metals as CNT–Cu interfacial interaction enhancer:*** Another strategy to improve CNT–Cu interaction is to add metals that easily form carbides to the CNT–Cu interface. The carbide-forming metals bridge CNTs with Cu because of their high affinity to carbon and ability to alloy with Cu. Several theoretical and experimental studies demonstrate the benefits of carbide-forming metals (iron [95], nickel [23,72,96–99], chromium [72,84,100–102], molybdenum [103], titanium [104], ruthenium [77], etc.) to improve CNT–Cu interfacial interaction. Milowska *et al*. [72] recommend using Ni, Cr and Al as interfacial enhancers to improve composite conductance by boosting CNT integration with Cu matrix. Their first principle calculations indicate that these metals create favourable interface geometries, increase density of states and reduce contact resistance. Similarly, molecular dynamics simulations predict significant improvements in Cu/CNT mechanical strength and damping characteristics with Ni as the interfacial enhancer [96,97]. This improvement is attributed to Ni–CNT and Ni–Cu attractions causing a strong adhesive force between the Cu matrix and nanotube filler (compared to the weak van der Waals Cu–CNT interactions in the absence of Ni). Experimentally, Lim *et al.* [98] and Kim *et al*. [99] have shown improved tribological properties in Cu/Ni/CNT versus Cu/CNT, while Nie *et al*.'s [103] Cu/Mo/CNT composites show improved electrical conductivity, thermal conductivity, tensile strength and hardness (than Cu/CNT). In some experimental studies, carbides are detected at the CNT/additive interface and are credited for improving interfacial bonding and Cu/CNT properties. Cheng *et al*. [104] added Ti as the interfacial enhancer and observed TiC formation at the CNT–Cu interface. Due to the crystallographic matching between the TiC (002) and Cu (002) planes, Cu/Ti/CNT composites showed better mechanical strength than Cu/CNT. In another work, Chu *et al*. [84] fabricated Cu/Cr/CNT composites with higher hardness and tensile strengths than Cu/CNT, which is credited to a thin Cr_3_C_2_ layer formed at the CNT–Cr interface.

Despite promise shown by additives in improving interfacial interaction between CNTs and Cu, composites with interfacial enhancers have not experimentally shown overall performances surpassing that of copper. This is because there is a lack of understanding on how additives affect CNT–Cu interface (and composite performance), and methods to regulate interfacial enhancers to improve composite properties are virtually non-existent. In terms of understanding the role of interfacial enhancers, benefits of additives have been demonstrated only in low CNT vol% composites, fabricated mainly by powder-processing. Literature on the effects and benefits of additives in high vol% (electrodeposited) Cu/CNT is missing and research efforts are required to fulfil this lacuna.

In some cases, additives are seen to show a trade-off between properties, improving one set of properties (usually mechanical), while degrading others (thermal and electrical). For example, in composites prepared by molecular mixing, oxygen inclusion degrades Cu/CNT thermal conductivity, while improving strength [94]. The poor thermal conductivity is attributed to carbon lattice damage at the CNT–Cu interface caused by nanotube functionalization applied for interfacial oxygen inclusion during Cu/CNT fabrication. To harness and understand the benefits of interfacial oxygen on composite properties, the negative effects of nanocarbon lattice damage need to be decoupled and suppressed. Toward this, we recommend exploring interfacial oxygen inclusion using alternative milder covalent and non-covalent CNT functionalization methods. As a precedent, Mendoza *et al*.'s composites [43] fabricated by molecular mixing using non-covalently functionalized (surfactant-wrapped) SWCNTs show an increase in both mechanical (hardness) and electrical properties (conductivity) without trade-off. However, in their study, the extent of oxygen inclusion at the interface is unclear as the elemental composition of the Cu–O–CNT interface is not explicitly characterized. In general, there is a lacuna of sufficient characterization data on Cu–additive–CNT interfaces. Also, studies determining the additive amount and Cu–additive–CNT bond type required to minimize property trade-off in composites are absent. Additional efforts need to be devoted toward rigorously characterizing the CNT–additive–Cu interface and identifying interface structures and compositions capable of enhancing Cu/CNT performances without trade-offs.

Besides sufficient CNT–Cu interface characterization, research efforts are also required to explore fabrication routes to control the amount, bonding type and location of interfacial enhancers in composites. In terms of location of interfacial enhancers, Milowska *et al*. [72] suggest that carbide-forming metal additives are to be included at CNT ends rather than on sidewalls to increase electrical properties. Current processing strategies are insufficient to achieve additive inclusion with such atomic-level precision. Also, studies similar to Milowska *et al*. on the effect of additive element's position on thermal and mechanical performances are necessary.

#### 3.1.3. Control CNT and Cu-matrix attributes

The composite performance is likely to be affected by the basic attributes of the two constituents i.e. CNTs (such as diameter, wall number, etc., figure 6*c*) and Cu matrix (grain size (figure 6*d*) and micro/nanostructures). However, concrete information on the ideal CNT and Cu attributes required to maximize overall composite performances is missing and systematic studies are required in this direction.

**(*a*) *Effects and control of CNT attributes in composites:*** The CNT wall number, diameter, length and purity affect the volume occupancy and interfacial area in the composites, influencing Cu/CNT performances. Further, since CNTs are one-dimensional nanostructures, their orientation in the composites relative to applied stress, heat and current flow directions are also likely to affect the Cu–CNT stress transfer as well as electron and phonon transport behaviours. Several studies explore the effects of these CNT attributes on composite properties and some examples are given below. Nayan *et al*. [28] and Guiderdoni *et al*. [32] show that the smaller the nanotube wall number, the better are the wear properties, hardness and mechanical strength. Both studies attribute their results to weak van der Waals forces between concentric walls of multiwall CNTs leading to lower shear resistances and poorer CNT–Cu interfacial strengths. Sun & Chen [105] show an analogous inverse trend for mechanical strength versus nanotube diameter, i.e. the smaller the nanotube diameter, the larger was the tensile strength due to the large total interfacial bonding area. A few studies have explored the effect of CNT length, mainly on Cu/CNT strength. Experimental results by Tsai & Jeng [45] indicate that shorter CNTs lead to higher strengths and stiffnesses in composites. Their simulations indicate that the CNT buckling behaviour in composites (responsible for strain release from the matrix) depends on the CNT length and that shorter CNTs lead to global buckling, while longer CNTs induce local buckling. However, Duan *et al*.'s [97] calculations suggest that when Cu–CNT interaction is through adhesive forces, the pull-out force is proportional to CNT length. Hence, for composites with interfacial enhancers, the longer the CNTs, the higher is the strength. The effect of CNT diameter, wall number and length on electrical and thermal properties is less well known. Shuai *et al*. [57] and Sundaram *et al*. [63] point out that the presence of CNT ends degrades composite CCC and electrical conductivity, implying that longer CNTs are preferred for high electrical performances.

The influence of CNT orientation on composite properties has been explored in both theoretical and experimental works. Computational studies by Ghorbani-Asl *et al*. [106] indicate that CNT alignment with applied bias is preferred for high Cu/CNT electrical conductivities. Experimentally, CNT orientation has been observed to affect performances of both powder-processed and electrodeposited composites. In powder-processed unidirectionally aligned CNT/Cu, maximum composite strength, electrical and thermal conductivities, as well as wear properties are observed along the CNT orientation direction, while maximum hardness is seen perpendicular to the CNT orientation [36–38]. In electrodeposited samples, Cu/CNT with unidirectionally aligned nanotubes [55] are observed to show higher strengths than copper along the CNT orientation. Besides orientation, CNT purity in terms of presence/absence of amorphous carbonaceous impurities (included during CNT synthesis) also critically affects Cu/CNT properties. Cho *et al*. [107] show that composite thermal conductivities decrease with inclusion of amorphous carbon impurities, which occupy the CNT–Cu interface and act as thermal barriers. Preliminary results in the current literature give a general idea that small-diameter, few-walled, pure, long and oriented CNTs may probably be beneficial for composite performances. However, exerting control over CNT attributes in Cu/CNT presents three difficulties.

First, a major gap in literature is the absence of experimental studies on how CNT crystallinity (measured as G/D) and chirality influence Cu/CNT performance. Ballistically conducting (i.e. metallic) nanotubes without defects (i.e. high G/D ratios) are predicted [79] to be required to achieve electrical conductivities double that of copper and high thermal conductivities [80,83]. There are few theoretical works on the effect of CNT chirality on composite performance, but none on G/D. With regard to nanotube chirality, Ghorbani-Asl *et al*. [106] show that the composite electrical conductance depends only weakly on nanotube chirality using non-equilibrium Green's function approach when the CNT–Cu interaction is poor. However, the impact of CNT chirality in composites with interface enhancers that allow for better CNT–Cu interaction are unknown. Both theoretical and experimental studies are required in this direction. In addition, modelling studies show that CNT chirality affects Al/CNT composite mechanical properties [108] and armchair CNTs enhance mechanical performances more than zig-zag CNTs. However, similar studies on Cu/CNT are missing and are necessary.

Second, even if the ideal CNT attributes for maximum composite performances are known, synthesizing and utilizing such CNTs for composite fabrication is inherently a major issue. CNT synthesis always yields materials with a distribution of lengths, diameters, wall numbers and chiralities. Making nanotube materials with a higher degree of structural control is a challenge to be addressed by the CNT synthesis research community. Specifically, chiral-selective CNT manufacture is a major problem currently tackled by the nanotube synthesis community [86,109,110].

Third, CNT attributes can change during composite fabrication processing. The issue is encountered especially in controlling length and orientation by mixing-based fabrications (mainly powder process) that break and shorten as well as misalign nanotubes. Modification of CNT attributes during composite fabrication also limits procuring reliable data on composite performance versus nanotube structure. However, recourses are available to minimize CNT structure modifications during composite fabrication. For example, Chen *et al*. used solution-based mixing methods to minimize CNT breakage while powder-processing Al-matrix/CNT composites and could precisely control nanotube aspect ratios on a wide range (6.5–55) to study strengthening effects [111]. Their results show that long CNTs (aspect ratio > 40) strengthened composites by load transfer, while shorter nanotubes strengthened composites by Orowan mechanism (i.e. CNTs loop and pin the dislocations). Similar alterations to powder-processing can be adopted for Cu/CNT fabrication. Further, more benign fabrications like template electrodeposition that preserve CNT attributes (length and orientation) during processing can also be used, especially for initial composite performance versus nanotube structure studies. The current nanotube synthesis technologies are sufficient to make templates with adequately controlled CNT diameter, wall number, G/D, orientation and purity for these initial studies [112–114].

**(*b*) *Effects and control of Cu-matrix attributes in composites***: Attributes of the Cu-matrix (grain size, micro/nanostructure, defect density, etc.) are highly likely to influence composite performances. Similar to conventional metals and alloys, Cu/CNT shows yield strength increase with decrease in grain size (grain refinement) in accordance with the Hall–Petch relationship. For instance, Kim *et al*. [115] demonstrate 27% increase in composite yield strength with drop in Cu grain size from 4 to 1.5 µm. This strengthening achieved by grain refinement is attributed to increased number of grain boundaries that pin dislocations, impede their motion and propagation, delaying the material's deformation and failure.

The Cu grain size is affected both by fabrication processing (powder method or electrodeposition) and by the CNTs themselves. In powder-processing, mainly the compaction stage affects the Cu grain size. Samples with average Cu grain sizes less than or equal to 100 nm have been obtained by severe plastic deformation processes, such as high-pressure torsion (HPT) and differential speed rolling [24,48–50,87,116,117]. On the other hand, sintering/hot pressing yield Cu grain sizes ranging from 100 nm to a few micrometres [43,115]. For electrodeposited composites, nucleation rates (a function of electrodeposition current, time, electrolyte concentration, etc.) affect grain attributes, and typical grain sizes range from 300 to 500 nm to a few micrometres [56,61,62,68,105].

Irrespective of the processing, CNTs by themselves aid refinement, decreasing average grain size and narrowing size distribution. Therefore, besides load-sharing, CNTs also contribute to mechanical property improvement by promoting grain refinement. Typically, grain size reductions greater than 50% are observed with CNT addition, and refinement increases with CNT vol% [24,39] in the absence of agglomeration. The mechanism by which CNTs cause grain refinement is fabrication-dependent. For example, in HPT processing, CNTs aid grain refinement by blocking and accumulating dislocations that eases sub-grain boundary formation (necessary for severe plastic deformation processes like HPT [117]). In sintering processes, CNTs located at grain boundaries restrain grain growth by the Zener pinning effect [39]. In electrodeposition, CNTs become nucleation centres and increase nucleation rate, leading to smaller grain sizes [68]. Besides affecting grain size, CNTs also increase dislocation density and twin-fault frequency in the Cu matrix [49,50].

While being a positive influence on mechanical properties, grain refinement is disadvantageous for composite electrical and thermal performances. Smaller grains, i.e. larger number of grain boundaries implies increased electron and phonon scattering at grain boundaries. Consequently, fine-grained composites have shown deteriorated electrical and thermal conductivities [39,62,118]. It is, however, possible to minimize this trade-off by micro/nanostructure tailoring, which has been demonstrated for pure copper. Lu *et al*. [119] achieved nanocrystalline pure copper samples with ultrahigh strength *and* high electrical conductivity by introducing a nanostructure consisting of multiple coherent twin boundaries. Similar efforts to determine Cu-grain size and structure regimes aimed at minimizing trade-off between electrical/thermal and mechanical properties resulting from grain refinement are necessary for Cu/CNT.

Tailoring Cu-matrix nano/microstructures may also be vital for improving Cu/CNT current stability and electromigration reliability for interconnect applications, as Cu diffusion is already restrained by nanotubes in the composites [3]. Composites with Cu-grain boundaries tailored perpendicular to current flow (i.e. bamboo micro/nanostructures) may show higher electromigration lifetimes and reliabilities than randomly oriented polygranular micro/nanostructures, especially in downsized configurations [120]. While the merit of tailored micro/nanostructures has been demonstrated for pure Cu interconnects, similar studies for Cu/CNT interconnects are necessary [121].

The main issues in regulating Cu-matrix attributes to maximize overall composite performances are summarized below:

(i) lack of complete understanding on how Cu-matrix attributes influence various composite properties and
(ii) lack of methods to control Cu-matrix attributes.

In terms of gaining understanding, the shortage of data on Cu-matrix attributes versus composite electrical/thermal properties needs to be addressed. Specifically, studies on CTE, CCC, and TCR versus Cu grain size and micro/nanostructure are missing and are required. Further, in the extant literature, grain-size effects have always been intertwined with CNT vol% and agglomeration effects. Therefore, exclusive studies on grain-size effects on electron and phonon transport are needed in homogeneous composites. On a related note, composite performance related to Cu grain sizes are more widely reported for low CNT vol% samples, usually obtained by powder-processing. Analogous studies are essential for high CNT vol% composites, especially those obtained by electrodeposition.

Finding routes to exercise control on Cu grain size and micro/nanostructure in Cu/CNT needs further research on fabrication methods. Most studies on Cu grain size control so far have focused on powder-processing, and regulation of Cu-matrix attributes by electrodeposition needs exploration. For electrodeposition, as mentioned earlier, Cu grain size and distribution are dictated by Cu nucleation and growth rates known to depend on parameters such as electrolyte concentration, current, time, etc. [56,61,62,68,105]. The interconnects industry has already evolved methods, such as using organic additives, altering electrodeposition parameter, and post-fabrication processing for Cu grain control, which can be borrowed and tested for Cu/CNT preparation [122–125].

In addition, further research is required to understand how CNT attributes (defects, diameter, wall number, orientation, etc.) affect Cu-matrix characteristics. With this understanding, the CNTs themselves can be used as tools for regulating Cu grain size and micro/nanostructures. A few reports suggest that CNT orientation and functionalization influence Cu grain sizes. For example, samples with cross-plied CNTs have been observed to show smaller grain and twin lamellae sizes than those with unidirectionally oriented CNTs [62]. In another study, functionalized CNTs were seen to result in smaller Cu grains than non-functionalized CNTs [61]. However, these studies have been only on electrodeposited Cu/CNT. In a CNT wall number versus Cu/CNT performance study, Guiderdoni *et al*. [32] did not measure Cu grain sizes due to composite inhomogeneity and wide grain-size distribution in their samples compacted by SPS. Therefore, studies on how CNT attributes affect Cu-matrix attributes are necessary on homogeneous powder-processed composites.

### 3.2. Cu/CNT industrialization and real-world application as Cu-substitutes

We can conclude that Cu/CNT has real-world application potential, primarily as a Cu substitute, based on properties demonstrated in literature (§2) and avenues available for performance enhancement (§3.1). However, practical Cu/CNT application presents several issues depending on the market, i.e. niche or mainstream.

#### 3.2.1. Cu/CNT for niche applications

Using Cu/CNT in niche applications is a more realistic beginning, as market barriers are lower than in mainstream applications, i.e. the mass-production and cost requirements are low. Niche markets tolerate disruptive technologies and simply aim to exploit the advantages of Cu/CNT over Cu in terms of properties for highly specific applications. One example of a niche market for Cu/CNT is the high-end electronics industry focusing on high-functionality high-power devices. High-end electronics demand special interconnects, thermal interface materials (TIMs) with heat and current stabilities greater than Cu and electrical and thermal conductivities at par with Cu. Even in the current state of development, Cu/CNT meets these requirements. For example, two-stage electrodeposited planar and vertical Cu/CNT interconnects show CCC greater than Cu, CTE and TCR less than Cu, as well as electrical and thermal conductivities similar to Cu. As an added advantage, the electrodeposition-based fabrication is electronics industry-compatible. To enable Cu/CNT application in high-end electronics, market analysis facilitated by industry–academia collaborations is required to identify material requirements (dimensions, properties, configurations, etc.) for specific device components. Further, Cu/CNT's application to high-end electronics can be accelerated by improving composite properties using suggestions listed in §3.1.

Besides replacing Cu (as a disruptive technology), Cu/CNT can fulfil applications envisaged for neat-CNT materials because of their better functionality and integrability. This part-constructive characteristic of Cu/CNT removes the major roadblock encountered by the CNT industry in finding real-world applications for nanotubes. For example, Cu/CNT could be a better choice than CNT materials as an electrode material in energy conversion, harvesting and storage systems. Luo *et al*. [82] demonstrate that quantum-dot-sensitized solar cells with Cu/CNT film counter electrodes show higher power conversion efficiency than neat CNT films (and conventional materials like platinum).

#### 3.2.2. Cu/CNT for mainstream applications

The two key mainstream applications of Cu/CNT as Cu-replacements are

(a) as interconnects and thermal interface materials in conventional electronics (cell phones, computers, etc.)
(b) as lightweight macroscopic conductors, such as wires/cables for motors, data and electricity transmission lines, etc.

Besides attaining composite performances better than Cu, mainstream application of Cu/CNT needs industrialization, i.e. reproducible mass-production at low cost using industry-compatible methods, while preserving properties. Cu/CNT industrialization for electronics and macroscopic conductors present different issues.

**(*a*) *Cu/CNT for mainstream electronics:*** For the electronics industry, electrodeposition-based Cu/CNT fabrication methods are most compatible. Co-electrodeposition is closest to the current processing technologies used by the electronics industry (damascene process [122–125]). However, co-electrodeposition entails several Cu/CNT structure control issues that need solving, such as obtaining individually dispersed long CNTs for homogeneous composite fabrication, controlling nanotube alignment, etc. In this light, CNT-template electrodeposition, which affords easier Cu/CNT structure control (with regard to composite homogeneity, CNT orientation, etc.) shows more promise than co-electrodeposition. Currently, maximum Cu/CNT electrical and thermal performances are achieved by template electrodeposition (two-stage process). However, current template-making methodologies for electrodeposition involve aligned CNT synthesis and transfer [3,6,59,60], which are expensive and unsuitable for mass-production. To address this, printing technologies to manufacture CNT templates for Cu electrodeposition need to be developed.

**(*b*) *Cu/CNT for lightweight macroscopic conductors:*** Mass-production technologies compatible with the established copper industry are highly desirable for composites to be used as lightweight conductors in electrical wiring, data cables, etc. Scale-up demonstrations for macroscopic Cu/CNT are already reported in literature [30,54,63]. For example, Xu *et al*. [54] demonstrate continuous fabrication of copper-coated CNT wires by template electrodeposition at approximately 16 cm min^−1^ with promising electrical and mechanical properties. However, the production methodology is unsuited for the copper-wire industry, which typically uses processes like die-drawing and annealing. Arnaud *et al*. [30] report drawing Cu/CNT wires from SPS sintered composite cylinders, which is more consistent with the copper-wire industry. However, sintering-based methods are low-throughput batch processes and continuous processing is favoured for large-scale manufacture.

Considering the fit with conventional Cu industry [126,127], ideal macroscopic Cu/CNT manufacture methods for mainstream applications are co-electrodeposition or melt-processing. Cu/CNT co-electrodeposition is compatible with standard Cu electrorefining processes; however, as discussed earlier, co-electrodeposition entails composite structure control issues yet to be solved. Cu/CNT manufacture melt-processing is a major challenge considering the poor wetting between molten Cu and CNTs. Addressing this challenge, Shugart & Scherer [128,129] and Knych *et al*. [130] succeeded in melt-processing Cu/MWCNT (2 wt%) composites with uniform Cu–CNT distribution and electrical conductivities higher than the base copper material. Cu/CNT fabrication was accomplished by adding nanotubes to vigorously stirred molten copper exposed to an electrical current in inert gas atmosphere. However, Cu/CNT melt-processing is still in the early stages of development. Comprehensive composite performances (mechanical, thermal and other electrical properties) are yet to be characterized and aspects of structural control (CNT and Cu attributes) need investigation.

In addition to mass-production technology development, Cu/CNT metrology and quality assurance protocols meeting industrial standards need establishing. In terms of metrology, key properties valued by the electronics industry (thermal/current cycling and reliability [131]) and the copper wire conductors industry (alternating current and data transmission properties) need to be evaluated and reported. For Cu/CNT quality assurance, standard tests established for electronics and wire industries (prescribed by ASTM [132], IEC [133], IPC [134], IEEE (http://standards.ieee.org/index.html (accessed 18 May 2018)), etc.) can be applied. Applying standardized testing also facilitates comparison between Cu/CNT samples and versus copper, assisting both industrialization and research efforts.

## 4. Summary

In this paper, we trace the research and development of Cu/CNT and offer our perspectives on Cu/CNT's potential to meet demands for materials outperforming copper for next-generation applications. We discuss the emergence, achievements and limitations of the two major Cu/CNT fabrication approaches ((1) powder-processing and (2) electrochemical deposition). Powder-processing has produced composites superior to Cu mainly in terms of mechanical performances (strength, hardness, stiffness, etc.) due to the reinforcing effect of CNTs. However, the method precludes inclusion of greater than 20 vol% of CNTs (required for weight reduction) and composite structure control (in terms of size, shape, nanotube alignment, etc.). Overcoming these limitations, electrodeposition has achieved micro and macroscale composites with greater than 45 vol% CNTs (i.e. 34–80% lighter than Cu) with mechanical, thermal and electrical properties rivalling that of Cu. Specifically, electrodeposited composites show superior heat and current stabilities than Cu with at par electrical and thermal conductivities. These comprehensive high performances (versus copper) attest to Cu/CNT's potential as a future Cu alternative in electrical and thermal applications ranging from electronics to transmission wires and data cables.

For Cu/CNT to replace copper in real-life applications, two grand challenges need to be solved: (1) fabricating lightweight composites outperforming Cu in terms of electrical, mechanical and thermal properties and (2) industrializing Cu/CNT. To beat Cu performances (grand challenge 1), the real-world composites need to get closer to the ideal composite (i.e. Cu matrix embedding a *high vol% of uniformly distributed aligned individual CNTs*), theoretically predicted to show properties better than Cu. We specify three issues to be addressed by the research community to nudge real-world composites closer to the ideal: (i) achieve uniform CNT–Cu distribution at high CNT vol%, (ii) ensure effective CNT–Cu interaction, and (iii) control CNT and Cu-matrix attributes. We discuss practical avenues to address each of these issues in light of efforts already reported in literature. Transferring Cu/CNT from the lab bench to real-life applications (grand challenge 2) presents market-dependent issues, i.e. niche or mainstream. Niche applications (such as high-end interconnects, TIMs, etc.) with a high tolerance to disruptive technologies and lower market barriers are a more realistic terrain for initial Cu/CNT industrialization. Encouragingly, currently available microscale Cu/CNT, with their current- and heat-stability properties better than Cu and device-industry-friendly fabrication, already qualify for application in high-end interconnects. Mainstream application of Cu/CNT in conventional electronics (as interconnects and TIMs) and as lightweight macroscopic conductors (cables/wires for motors, data and electrical transmission, etc.) requires reproducible mass-production at low cost, while attaining the benchmark properties. The Cu/CNT mass-production technology needs to be compatible with existing industrial practices. While the mainstream electronics require cheap CNT template-making and electrodeposition protocols, mass-production of Cu/CNT macroscopic conductors needs to be aligned with traditional Cu-industry methods, such as co-electrodeposition and melt-processing. Efforts to develop Cu/CNT manufacturing methodologies viable for mainstream applications have already begun. Replacing the ubiquitous copper with high-performance Cu/CNT translates to more powerful and sustainable future electrical and electronics systems, i.e. more fuel-efficient transportation and smaller devices with higher functionality. With continued efforts from academia and industry, we expect real-life application of Cu/CNT as a lighter and superior-performing Cu substitute to be realized with immense positive impact to our everyday lives.

## Supplementary Material

Raw data of Fig. 4A

## Supplementary Material

Raw data of Fig. 4C

## Supplementary Material

Raw data of Fig. 4D

## Data accessibility

Datasets corresponding to figure 4 (*a*, *c* and *d*) have been uploaded in the electronic supplementary material.
